# Cost-effectiveness of interferon-*γ* release assay for screening of latent tuberculosis infection in individuals with schizophrenia

**DOI:** 10.1017/S0950268823002030

**Published:** 2024-01-05

**Authors:** Akiko Kowada

**Affiliations:** Department of Occupational Health, Kitasato University Graduate School of Medical Sciences, Sagamihara, Kanagawa, Japan

**Keywords:** health economics, interferon-γ release assay, rifampin, schizophrenia, tuberculosis prevention

## Abstract

Schizophrenia is recognized as a significant risk factor for tuberculosis (TB). This study aimed to evaluate the effectiveness and cost-effectiveness of interferon-γ release assay (IGRA) with preventive treatment for screening of latent tuberculosis infection (LTBI) in individuals with schizophrenia. A state transition model was developed from a healthcare payer perspective on a lifetime horizon. Ten strategies were compared by combining two different tests for LTBI, i.e. IGRA and tuberculin skin test (TST), and five different preventive treatments, i.e. 9-month isoniazid (9H), 3-month isoniazid and rifapentine (3HP) by directly observed therapy, 3HP by self-administered therapy, 3-month isoniazid and rifampin (3RH), and 4-month rifampin (4R). The main outcomes were costs, quality-adjusted life-years (QALYs), life expectancy life-years (LYs), incremental cost-effectiveness ratios, drug-sensitive tuberculosis (DS-TB) cases, and TB-related deaths. For both bacillus Calmette–Guérin (BCG)-vaccinated and non-BCG-vaccinated individuals, IGRA with 4R was the most cost-effective and TST with 3RH was the least effective. Among schizophrenic individuals in Japan, IGRA with 4R saved US$17.8 million, increased 58,981 QALYs and 935 LYs, and prevented 222 DS-TB cases and 75 TB-related deaths compared with TST with 3RH. In individuals with schizophrenia, IGRA with 4R is recommended for LTBI screening with preventive treatment to reduce costs, morbidity, and mortality from TB.

## Introduction

Tuberculosis (TB) remains a global public health threat and was the leading cause of death from a single infectious agent until the coronavirus (COVID-19) pandemic, with 6.4 million new cases of TB diagnosed in 2021 [1]. Japan, one of the low-burden countries, had a notification rate of 9.2 cases per 100,000 population for all TB cases, and 11,519 new cases of TB were diagnosed in 2021 [2].

Schizophrenia affects approximately 24 million people worldwide and 1 million in Japan [3, 4]. Schizophrenia is associated with a weighted average of 14.5 years of potential life loss [5]. The incidence and mortality of TB in schizophrenic patients are significantly higher than those in the general population [6, 7]. Overcrowded environments, poor communication skills, high levels of substance abuse such as smoking and alcohol use, poor adherence to medications, hidden comorbidity of lifestyle diseases from unhealthy lifestyle habits such as diabetes, hypertension, cardiovascular disease, cancer, and dyslipidemia [8], and low socio-economic status not only delay the diagnosis and treatment of TB but also spread TB infection in public welfare facilities and psychiatric hospitals, leading to TB outbreaks. An effective and integrated preventive care approach to TB in schizophrenic patients is urgently needed to improve welfare and public health.

For screening of latent tuberculosis infection (LTBI), *Mycobacterium tuberculosis*-specific interferon-γ release assay (IGRA) provides a more accurate diagnosis of TB infection than the tuberculin skin test (TST), especially in the bacillus Calmette–Guérin (BCG)-vaccinated individuals, because IGRA is not affected by BCG vaccination and has high specificity. For treatment of LTBI, there are several short-course preventive treatment regimens, i.e. once-weekly rifapentine 900 mg plus isoniazid 900 mg for 3 months (3HP) by directly observed therapy (DOT) or self-administered therapy (SAT), daily self-administered rifampin 600 mg for 4 months (4R), and daily self-administered isoniazid 300 mg with rifampin 600 mg for 3 months (3RH), which are preferred over longer-course (6- to 9-month) daily isoniazid monotherapy [9]. Longer-course isoniazid monotherapy is efficacious but has a higher toxicity risk and lower treatment completion rates than shorter rifamycin-based regimens [10–12]. The effective strategy combining screening and treatment of LTBI has the potential to reduce morbidity and mortality in people with schizophrenia.

Cost-effectiveness studies considering the costs and the benefits of IGRA and short-course preventive treatment regimens for LTBI in individuals with schizophrenia warrant evaluation.

This study aimed to evaluate the effectiveness and cost-effectiveness of IGRA with preventive treatment for screening of LTBI in individuals with schizophrenia.

## Methods

### Study design

The economic evaluation was conducted based on a state transition model from a healthcare payer perspective on a lifetime horizon. Each cycle length was one year to correspond with the typical duration for TB screening. The half-cycle correction was applied. Decision branches led directly to one Markov node for each intervention strategy, and the initial event was modelled within a Markov cycle tree. The target population was a hypothetical cohort of individuals with schizophrenia aged 43 years, the average age of schizophrenic patients [13, 14]. Ten strategies were compared by combining two different tests for LTBI, i.e. IGRA and TST, and five different preventive treatments, i.e. 9-month isoniazid (9H), 3HP by DOT, 3HP by SAT, 3RH, and 4R [15]. The discounting annual rate for costs and health state utility values was fixed at 3% [16]. The main outcome measures were costs, quality-adjusted life-years (QALYs), life expectancy life-years (LYs), incremental cost-effectiveness ratios (ICERs), drug-sensitive tuberculosis (DS-TB) cases, and TB-related deaths. The willingness-to-pay (WTP) threshold was US$50,000 per QALY gained [17].

The model was constructed with TreeAge Pro 2023 (TreeAge Software, Williamstown, MA). This study is reported following the Consolidated Health Economic Evaluation Reporting Standards 2022 (CHEERS 2022) statement [18]. Ethics approval was not required because this was a modelling study with all inputs and parameters derived from the published literature and Japanese statistics.

### Model structure

In an LTBI screening strategy, individuals with schizophrenia underwent LTBI diagnostic tests: IGRA or TST. All individuals with a positive LTBI diagnostic test received a chest X-ray examination. If TB was detected on the chest X-ray, the individuals had smear and culture tests of sputum and drug sensitivity tests and were treated according to the standard TB treatment protocol. If TB was ruled out by chest X-ray, the individuals received preventive treatment. After initiating the preventive treatment, the individuals either completed treatment in full or discontinued treatment due to serious adverse events of preventive treatment. Those with a negative LTBI diagnostic test did not receive preventive treatment.

### Model input and assumption

Clinical probabilities were collected using MEDLINE and Japanese statistics from 1980 to November 2023, to estimate input parameters for the models (Table 1). The BCG vaccination rate was 98% in Japan [19]. Sensitivities and specificities of IGRA, TST, and chest X-ray were obtained from systematic reviews and the literature [20–22]. Mortality due to other causes in people with schizophrenia was estimated from the literature and Japanese vital statistics [23, 24]. LTBI prevalence, TB incidence, and DS-TB mortality in a schizophrenic cohort were estimated from Japanese TB statistics and the literature [2, 7, 25, 26]. Multi-drug-resistant tuberculosis (MDR-TB) rate, TB recurrence rate, the mortality of untreated TB, and the annual risk of TB reactivation were obtained from Japanese TB statistics and the literature [2, 27, 28]. Efficacy, completion rates, and adverse event rates of five preventive treatments were obtained from the literature [10–12, 29–32].

**Table 1.** Baseline estimates for selected variables

Variable	Baseline value	One-way sensitivity analysis range	Probabilistic sensitivity analysis distribution	References
LTBI prevalence in individuals with schizophrenia aged 43 years	0.08	0.01–0.2		[7, 25]
TB incidence in individuals with schizophrenia aged 43 years	0.000061	0.00001–0.0001		[2, 7]
MDR-TB rate	0.007	0.001–0.04	Beta	[2]
Recurrence rate of DS-TB	0.041	0.01–0.1	Beta	[2]
Recurrence rate of MDR-TB	0.02	0.01–0.1	Beta	[2]
Annual risk of TB reactivation with negative TST or IGRA results	40 years 0.0021	N/A		[28]
	50 years 0.0015			
	60 years 0.0012			
Annual risk of TB reactivation with positive TST or IGRA results	40 years 0.0035	N/A		[28]
	50 years 0.0035			
	60 years 0.0016			
Mortality of DS-TB in individuals with schizophrenia aged 43 years	0.061	0.01–0.5		[2, 26]
Mortality due to other causes in individuals with schizophrenia aged 43 years	0.0045	0.001–0.01		[23, 24]
Mortality of MDR-TB	0.408	0.2–0.6	Beta	[2, 26]
Mortality of untreated TB	0.70	0.53–0.86	Beta	[27]
Efficacy of preventive treatment	0.90	0.63–0.97	Beta	[10, 29, 30]
Completion rate of 9H	0.63	0.3–0.9	Beta	[10, 12]
Completion rate of 3HP by DOT	0.87	0.3–0.9	Beta	[11, 12]
Completion rate of 3HP by SAT	0.74	0.3–0.9	Beta	[11, 12]
Completion rate of 4R	0.79	0.3–0.9	Beta	[10, 12]
Completion rate of 3RH	0.72	0.3–0.9	Beta	[12, 31]
Adverse event rate of 9H	0.049	0.01–0.1	Beta	[10]
Adverse event rate of 3HP by DOT	0.036	0.01–0.1	Beta	[11]
Adverse event rate of 3HP by SAT	0.056	0.01–0.1	Beta	[11]
Adverse event rate of 4R	0.026	0.01–0.1	Beta	[10]
Adverse event rate of 3RH	0.049	0.01–0.1	Beta	[32]
BCG vaccination rate	0.98	0.8–1	Beta	[19]
Sensitivity of TST (%)	77	71–82	Beta	[20]
Specificity of TST, non-BCG (%)	97	95–99	Beta	[20]
Specificity of TST, BCG (%)	59	46–73	Beta	[20]
Sensitivity of IGRA (%)	93	86–96	Beta	[21]
Specificity of IGRA (%)	98	94–99	Beta	[21]
Sensitivity of chest X-ray (%)	87.7	83.2–91.4	Beta	[22]
Specificity of chest X-ray (%)	64.3	62.1–66.4	Beta	[22]
Costs (US$)				
IGRA	58.2	43.7–72.8	Gamma	[33, 34]
TST	17.9	13.4–22.4	Gamma	
Chest X-ray	28.1	21.1–35.1	Gamma	
Smears, cultures, and drug sensitivity tests of sputum examination	193.7	145.3–242.1	Gamma	
9H	348.2	261.2–435.3	Gamma	
3HP by DOT	863.1	647.3–1078.9	Gamma	
3HP by SAT	310.5	232.9–388.1	Gamma	
4R	246.4	184.8–308.0	Gamma	
3RH	230.1	172.6–287.6	Gamma	
Treatment of adverse events	64.4	48.3–80.5	Gamma	
Standard treatment of DS-TB	12,559	9,419–15,699	Gamma	
Treatment of MDR-TB	113,320	84,990–141,650	Gamma	
TB-related death	31,302	23,477–39,128	Gamma	[35]
Death	50,731	38,048–63,414	Gamma	[35]
Health state utility				
Stable	0.89	0.8–1.0	Gamma	[36]
LTBI	0.81	0.7–0.9	Gamma	[37]
LTBI with adverse events	0.61	0.5–0.7	Gamma	[34]
DS-TB	0.69	0.6–0.8	Gamma	[37]
MDR-TB	0.58	0.5–0.7	Gamma	
Dead	0	N/A		

3HP, once-weekly rifapentine 900 mg plus isoniazid 900 mg for 3 months; 3RH, daily self-administered isoniazid 300 mg with rifampin 600 mg for 3 months; 4R, daily self-administered rifampin 600 mg for 4 months; 9H, daily self-administered isoniazid 300 mg for 9 months; BCG, bacillus Calmette–Guérin; DOT, directly observed therapy; DS-TB, drug-sensitive tuberculosis; IGRA, interferon-γ release assay; LTBI, latent tuberculosis infection; MDR-TB, multi-drug-resistant tuberculosis; N/A, not applicable; SAT, self-administered therapy; TB, tuberculosis; TST, tuberculin skin test.

The model applied a healthcare payer perspective and considered direct medical costs. All direct medical costs were adjusted to the 2021 Japanese yen, using the medical care component of the Japanese consumer price index, and were converted to US dollars, using the Organisation for Economic Co-operation and Development (OECD) purchasing power parity rate in 2021 (US$1=¥102.05) (Table 1) [33–35].

**Figure 1.**
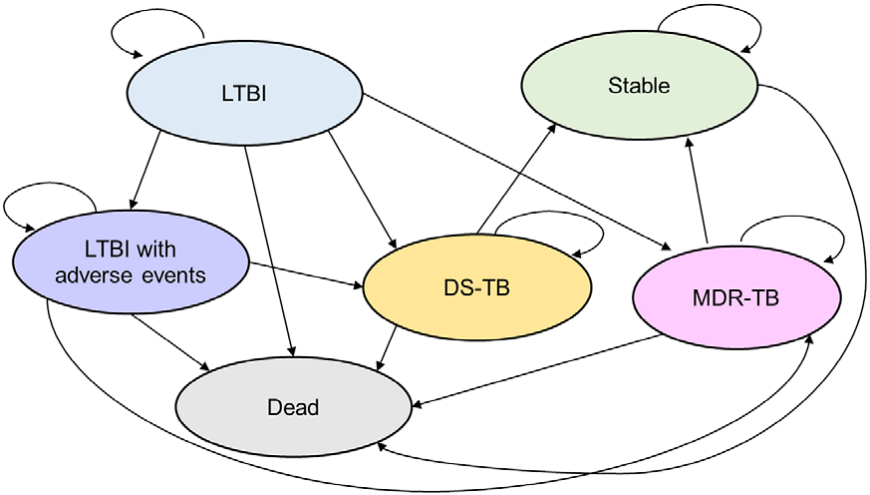
Schematic depiction of a Markov cycle tree in a state transition model. This figure displays the health states in the model as ovals, and the possible transitions between one health state and another during a one-year model cycle are indicated by arrows. DS-TB, drug-sensitive tuberculosis; LTBI, latent tuberculosis infection; MDR-TB, multi-drug-resistant tuberculosis.

Figure 1 illustrates the six key health states and possible transitions between them during each cycle in a Markov model to represent the possible clinical health states in the target population: (i) stable, (ii) LTBI, (iii) LTBI with adverse events, (iv) DS-TB, (v) MDR-TB, and (vi) dead. Health state utility values were obtained from the literature [34, 36, 37].

### Scenario analysis

A scenario analysis was applied to simulate non-BCG vaccination status as applied in the United States and some European low-TB-incidence countries.

### Sensitivity analyses

One-way sensitivity analyses were performed to examine the effect of changes in parameter values, including LTBI prevalence in individuals with schizophrenia; TB incidence in individuals with schizophrenia; MDR-TB rate; the recurrence rate of DS-TB and MDR-TB; mortality of MDR-TB and DS-TB; efficacy of preventive treatment; completion rate of preventive treatment; adverse event rate of five preventive treatments; BCG vaccination rate; sensitivity and specificity of TST, IGRA, and chest X-ray; health state utilities; the cost of LTBI screening, five preventive treatments, treatment of DS-TB and MDR-TB, TB-related death, and death. The ranges of sensitivity analysis for the variables are presented in Table 1. A wide range of sensitivity analyses for completion rates (0.3 to 0.9) and adverse event rates (0.01 to 0.1) of five preventive treatments were considered to make the variation intervals of all completion rates realistic. The ICER tornado diagram was shown to evaluate the impact of the variation of each parameter between the two LTBI screening strategies. Probabilistic sensitivity analyses using a second-order Monte Carlo simulation during 10,000 reiterations were conducted to assess the effect of parameter uncertainties on the base case estimates. The variables included in the probabilistic sensitivity analyses are listed in Table 1, along with the type of distribution. The uncertainty had a beta distribution for probability, health state utility, and accuracy, and a gamma distribution for cost.

### Markov cohort analyses and cumulative lifetime health outcomes

Markov cohort analyses were performed to obtain the cumulative lifetime probability of DS-TB, and TB-related deaths by each LTBI screening strategy. To estimate the cumulative lifetime number of DS-TB cases and TB-related deaths for each LTBI screening strategy, I multiplied the cumulative lifetime probability of DS-TB and TB-related deaths for each LTBI screening strategy by the total number of individuals with schizophrenia. The total number of schizophrenia patients in Japan in 2017 was estimated at 1,002,000 [4]. The numbers of DS-TB cases prevented and TB-related deaths averted by each LTBI screening strategy compared with the least effective LTBI screening strategy were calculated by subtracting those numbers for each LTBI screening strategy from those numbers for the least effective LTBI screening strategy, respectively.

## Results

In the base case analysis, IGRA with 4R was the least expensive and most effective (US$221,197, 16.02489 QALYs, 18.13834 LYs). TST with 3RH was the least effective (US$221,215, 15.96603 QALYs, 18.13740 LYs) (Table 2). For non-BCG-vaccinated individuals with schizophrenia in the scenario analysis, IGRA with 4R was more cost-effective (US$221,197, 16.02489 QALYs, ICER, US$36,893 per QALY gained, 18.13834 LYs) than TST with 4R (US$221,159, 16.02386 QALYs, 18.13826 LYs) and other strategies (Table 2).

**Table 2.** Results of base case and scenario analyses

Strategy	Costs (US$)	Incremental cost (US$)	QALYs	Incremental QALYs	ICER (US$/QALY gained)	LYs
Base case						
TST with 3RH	221,215		15.96603			18.13740
TST with 9H	221,241	26	15.96670	0.00067	38,806	18.13749
TST with 3HP by SAT	221,231	Saving	15.97439	0.00769	Dominant	18.13794
TST with 3HP by DOT	221,358	127	15.98253	0.00814	15,602	18.13858
TST with 4R	221,212	Saving	15.99556	0.01303	Dominant	18.13826
IGRA with 3HP by SAT	221,210	Saving	16.02099	0.02543	Dominant	18.13795
IGRA with 3RH	221,209	Saving	16.02157	0.00058	Dominant	18.13787
IGRA with 9H	221,225	16	16.02203	0.00046	34,783	18.13740
IGRA with 3HP by DOT	221,216	Saving	16.02306	0.00103	Dominant	18.13872
IGRA with 4R	221,197	Saving	16.02489	0.00183	Dominant	18.13834
Scenario (non-BCG vaccination status)						
TST with 3RH	221,169		16.00519			18.11417
TST with 3HP by SAT	221,171	2	16.01824	0.01305	153	18.13794
TST with 9H	221,183	12	16.02003	0.00179	6,704	18.13749
IGRA with 3HP by SAT	221,210	27	16.02099	0.00096	28,125	18.13795
IGRA with 3RH	221,209	Saving	16.02157	0.00058	Dominant	18.13787
TST with 3HP by DOT	221,182	Saving	16.02171	0.00014	Dominant	18.13858
IGRA with 9H	221,225	43	16.02203	0.00032	134,375	18.13740
IGRA with 3HP by DOT	221,216	Saving	16.02306	0.00103	Dominant	18.13872
TST with 4R	221,159	Saving	16.02386	0.00080	Dominant	18.13826
IGRA with 4R	221,197	38	16.02489	0.00103	36,893	18.13834

3HP, once-weekly rifapentine 900 mg plus isoniazid 900 mg for 3 months; 3RH, daily self-administered isoniazid 300 mg with rifampin 600 mg for 3 months; 4R, daily self-administered rifampin 600 mg for 4 months; 9H, daily self-administered isoniazid 300 mg for 9 months; dominant, more effective and cost saving than other; DOT, directly observed therapy; ICER, incremental cost-effectiveness ratio; IGRA, interferon-γ release assay; LY, life expectancy life-year; QALY, quality-adjusted life-year; SAT, self-administered therapy; TST, tuberculin skin test.

**Figure 2.**
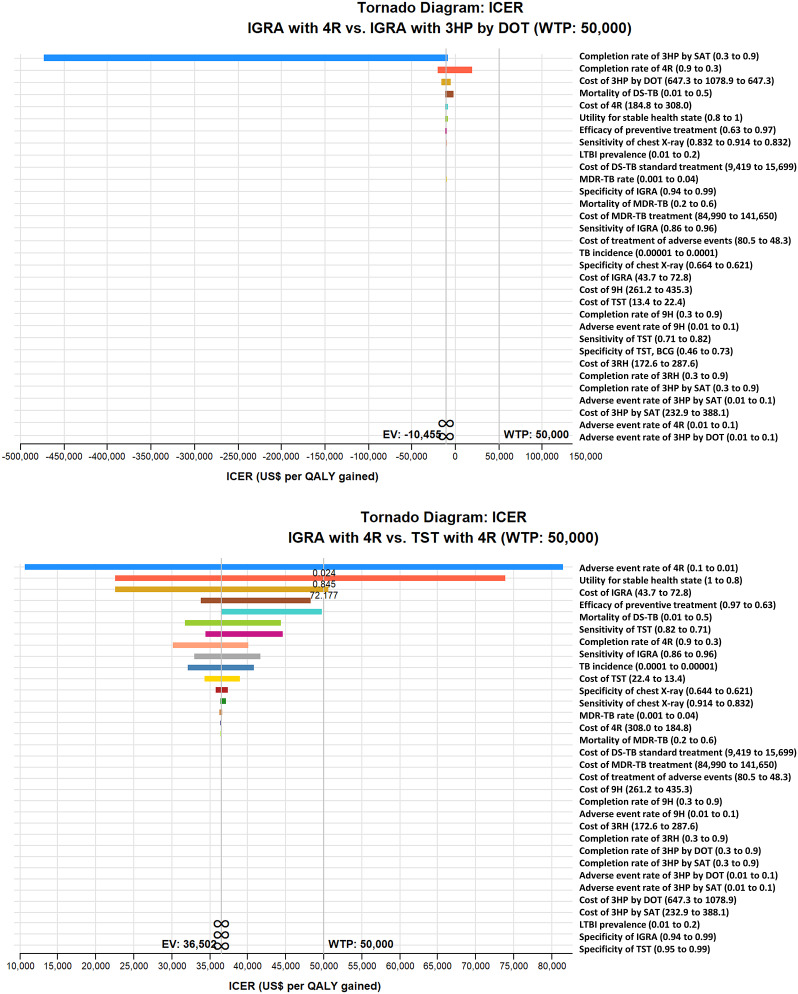
ICER tornado diagram. (a) IGRA with 4R versus IGRA with 3HP by DOT in schizophrenic individuals with a 98% BCG vaccination rate. (b) IGRA with 4R versus TST with 4R in non-BCG-vaccinated schizophrenic individuals. 3HP, once-weekly rifapentine 900 mg plus isoniazid 900 mg for 3 months; 3RH, daily self-administered isoniazid 300 mg with rifampin 600 mg for 3 months; 4R, daily self-administered rifampin 600 mg for 4 months; 9H, daily self-administered isoniazid 300 mg for 9 months; BCG, bacillus Calmette–Guérin; DOT, directly observed therapy; DS-TB, drug-sensitive tuberculosis; EV, expected value; ICER, incremental cost-effectiveness ratio; IGRA, interferon-γ release assay; LTBI, latent tuberculosis infection; MDR-TB, multi-drug-resistant tuberculosis; QALY, quality-adjusted life-year; SAT, self-administered therapy; TB, tuberculosis; TST, tuberculin skin test; WTP, willingness-to-pay.

Cost-effectiveness was not sensitive to any variable in schizophrenic individuals with a 98% BCG vaccination rate (Figure 2a), while in non-BCG-vaccinated schizophrenic individuals, cost-effectiveness was sensitive to the adverse event rate of 4R, the health state utility of stable individuals with schizophrenia, and the cost of IGRA (Figure 2b). When the adverse event rate of 4R was lower than 0.024, the health state utility of stable individuals with schizophrenia was lower than 0.845, and the cost of IGRA was higher than US$72.2, TST with 4R was more cost-effective than IGRA with 4R (Figure 2b).

**Figure 3.**
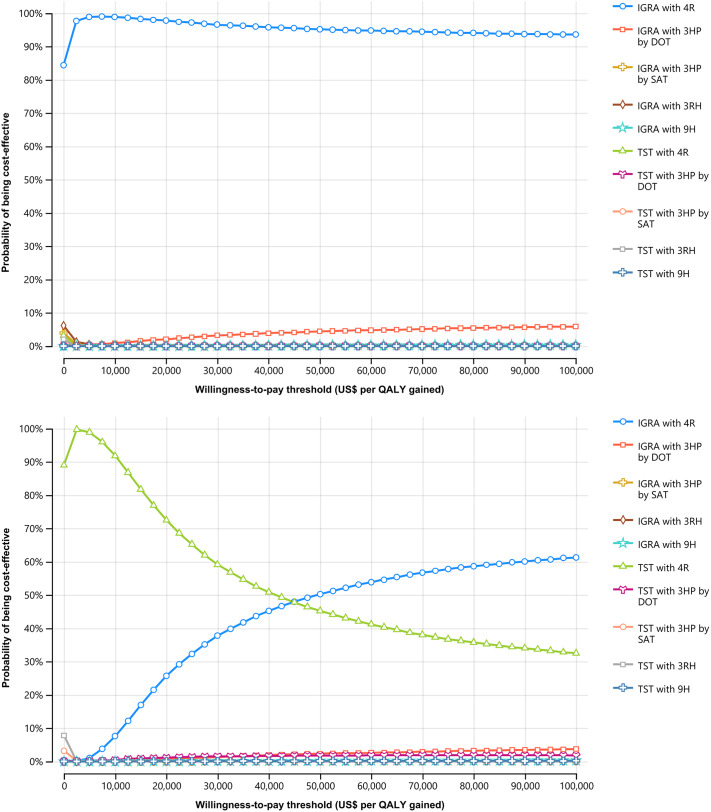
Cost-effectiveness acceptability curve. (a) Schizophrenic individuals with a 98% BCG vaccination rate. (b) Non-BCG-vaccinated schizophrenic individuals. 3HP, once-weekly rifapentine 900 mg plus isoniazid 900 mg for 3 months; 3RH, daily self-administered isoniazid 300 mg with rifampin 600 mg for 3 months; 4R, daily self-administered rifampin 600 mg for 4 months; 9H, daily self-administered isoniazid 300 mg for 9 months; BCG, bacillus Calmette–Guérin; DOT, directly observed therapy; IGRA, interferon-γ release assay; QALY, quality-adjusted life-year; SAT, self-administered therapy; TST, tuberculin skin test.

Cost-effectiveness acceptability curves demonstrated that IGRA with 4R was 95.1 and 50.2% cost-effective for schizophrenic individuals with a 98% BCG vaccination rate and non-BCG vaccination at a WTP threshold of US$50,000 per QALY gained (Figure 3a,b).

Among schizophrenic individuals in 2017, IGRA with 4R saved US$17.8 million, increased 58,981 QALYs and 935 LYs, and prevented 222 DS-TB cases and 75 TB-related deaths compared with TST with 3RH over a lifetime (Table 3).

**Table 3.** Cumulative lifetime economic and health impacts of LTBI screening strategies in individuals with schizophrenia

Strategy	Cost saving (costs) US$	QALY gain (QALYs)	LY gain (LYs)	DS-TB cases prevented (DS-TB cases) *n*	TB-related deaths averted (TB-related deaths) *n*
TST with 3RH	Ref (221,657,789,455)	Ref (15,997,962)	Ref (18,173,679)	Ref (3,376)	Ref (903)
TST with 9H	−26,255,280 (221,683,685,280)	669 (15,998,631)	84 (18,173,763)	−211 (3,587)	−55 (958)
TST with 3HP by SAT	−16,518,643 (221,673,948,643)	8,378 (16,006,340)	538 (18,174,217)	0 (3,376)	9 (894)
TST with 3HP by DOT	−143,239,091 (221,800,669,091)	16,529 (16,014,491)	1,178 (18,174,857)	437 (2,939)	115 (788)
TST with 4R	2,791,216 (221,654,638,784)	29,590 (16,027,552)	862 (18,174,541)	208 (3,168)	54 (849)
IGRA with 3HP by SAT	4,805,636 (221,652,624,364)	55,074 (16,053,036)	544 (18,174,223)	11 (3,365)	20 (883)
IGRA with 3RH	6,121,378 (221,651,308,622)	55,653 (16,053,615)	468 (18,174,147)	−30 (3,406)	9 (894)
IGRA with 9H	−10,088,537 (221,667,518,537)	56,111 (16,054,073)	−4 (18,173,675)	−284 (3,660)	−57 (960)
IGRA with 3HP by DOT	−1,383,070 (221,658,813,070)	57,145 (16,055,107)	1,317 (18,174,996)	428 (2,948)	129 (774)
IGRA with 4R	17,814,413 (221,639,615,587)	58,981 (16,056,943)	935 (18,174,614)	222 (3,154)	75 (828)

*Note:* The total number of schizophrenia patients in Japan in 2017 was estimated at 1,002,000 [4]. 3HP, once-weekly rifapentine 900 mg plus isoniazid 900 mg for 3 months; 3RH, daily self-administered isoniazid 300 mg with rifampin 600 mg for 3 months; 4R, daily self-administered rifampin 600 mg for 4 months; 9H, daily self-administered isoniazid 300 mg for 9 months; DOT, directly observed therapy; DS-TB, drug-sensitive tuberculosis; IGRA, interferon-γ release assay; LY, life expectancy life-year; QALY, quality-adjusted life-year; Ref, reference; SAT, self-administered therapy; TST, tuberculin skin test.

## Discussion

This study demonstrated that, for schizophrenic individuals, IGRA with 4R provides the greatest benefits with cost savings and has the potential to reduce the costs, morbidity, and mortality of TB. The cost-effectiveness superiority of IGRA is due to the higher specificity of IGRA over TST, which means that the IGRA strategy has fewer false positives and lower costs for screening than the TST strategy. The cost-effectiveness advantage of 4R among the five preventive treatments is due to the lower adverse event rate and the high completion rate of 4R.

Several cost-effectiveness studies have shown superior cost-effectiveness of short-course preventive treatment in low-TB-incidence countries. Holland et al. found that 4R is cost saving compared with 9H and that 3HP dominates all regimens at a relative risk of disease 5.2 times the baseline estimate, or with completion rates less than 34% for isoniazid or 37% for rifampin [34]. Shepardson et al. demonstrated that 3HP administered as a directly observed treatment may be a cost-effective alternative to 9H in the United States [38]. I have also previously shown that IGRA with 3HP yields the greatest benefits at the least costs compared with IGRA with 9H, TST with 3HP, TST with 9H, and chest X-ray [39]. However, there are no cost-effectiveness studies focused on patients with schizophrenia or mental disorders. To the best of my knowledge, this study is the first cost-effectiveness analysis in the world to evaluate LTBI screening with preventive treatment for individuals with schizophrenia.

There are several limitations. First, the completion rates and adverse event rates for preventive treatment in individuals with schizophrenia were estimated to be the same as in the general population. However, schizophrenic patients often have hepatic dysfunction and poor long-term medication compliance. To compensate for this uncertainty, I performed one-way sensitivity analyses of completion rates (0.3–0.9) and adverse event rates (0.01–0.1) over a wide range for the five preventive treatments, conducted probabilistic sensitivity analyses, and demonstrated the robustness of the results, especially in BCG-vaccinated individuals. Second, comorbidities such as diabetes, end-stage renal disease, and HIV were not considered in this study. Third, LTBI prevalence and TB incidence in individuals with schizophrenia were estimated from Japanese TB statistics and the literature in this study. Further research on the epidemiological input values of TB for schizophrenic patients is needed. Fourth, there is no gold-standard test for LTBI. Fifth, the annual risk of TB reactivation in individuals with schizophrenia was estimated to be the same as in the general population. However, the risk is expected to be much higher. Finally, there are different costs and medical systems in each country. Further cost-effectiveness studies with variation in each country are required.

In conclusion, IGRA with 4R is recommended for LTBI screening with preventive treatment to reduce costs, morbidity, and mortality from TB in individuals with schizophrenia. Introducing effective LTBI screening with preventive treatment to individuals with schizophrenia will not only prevent future TB and improve health outcomes including quality of life but also contribute to reducing global health inequalities.

## Data Availability

Details of the model, data inputs, and other assumptions are provided in the Methods section.

## Abbreviations

3HP: once-weekly rifapentine 900 mg plus isoniazid 900 mg for 3 months
3RH: daily self-administered isoniazid 300 mg plus rifampin 600 mg for 3 months
4R: daily self-administered rifampin 600 mg for 4 months
9H: daily self-administered isoniazid 300 mg for 9 months
BCG: bacillus Calmette–Guérin
DOT: directly observed therapy
DS-TB: drug-sensitive tuberculosis
ICER: incremental cost-effectiveness ratio
IGRA: interferon-γ release assay
LTBI: latent tuberculosis infection
LY: life expectancy life-year
MDR-TB: multi-drug-resistant tuberculosis
QALY: quality-adjusted life-year
SAT: self-administered therapy
TST: tuberculin skin test
WTP: willingness-to-pay
